# Qingyihuaji formula reverses gemcitabine resistant human pancreatic cancer through regulate lncRNA AB209630/miR-373/EphB2-NANOG signals

**DOI:** 10.1042/BSR20190610

**Published:** 2019-06-18

**Authors:** Peng Chen, Meiying Wang, Cuiping Wang

**Affiliations:** 1 Department of Traditional Chinese Medicine, Affiliated Hospital of Weifang Medical University, Weifang City 261031, China; 2 Department of Gastroenterology, Affiliated Hospital of Weifang Medical University, Weifang City 261031, China; 3 Department of Neurology, Affiliated Hospital of Weifang Medical University, Weifang City 261031, China

**Keywords:** EphB2, LncRNA AB209630, miR-373, NANOG, Pancreatic cancer, Qingyihuaji formula

## Abstract

To investigate the possible mechanism of Qingyihuaji formula (QYHJ) for reversing gemcitabine (GEM) resistant human pancreatic cancer. Cell proliferation, apoptosis, migration and invasion were detected in CFPAC-1 cells. Xenograft mice established with CFPAC-1 through subcutaneous on 33 immunodeficient nude mice and randomly divided into four groups: vehicle, GEM (35 mg/kg), QYHJ (40 g/kg), and GEM + QYHJ (35 mg/kg + 40 g/kg) groups for 28-day treatment. Tumor growth and the mRNA expression of lncRNA AB209630, miR373, EphB2, and NANOG evaluated in dissected tumor tissue by real-time PCR, the CD133+ cancer stem cells were isolated by flow cytometer, and the changes of the tumor sphere forming were measured. QYHJ, especially the combination of GEM and QYHJ, was significantly inhibited the cell proliferation and migration of CFPAC-1 *in vitro* in the indicated times. The combination of GEM and QYHJ also remarkably promoted the cell apoptosis of CFPAC-1. QYHJ treatment effectively blocked the tumor growth in nude mice. QYHJ, especially GEM + QYHJ treatment, was significantly increased the mRNA expression of lncRNA AB209630, significantly decreased the mRNA levels of miR373, EphB2 and NANOG, and markedly reduced the tumor sphere formation and the numbers of CD133+ stem cells. In addition, GEM alone treatment had no significant effect in the above biomarker changes. QYHJ could effectivly enhance the antihuman pancreatic tumor activity of GEM, which may be through inhibiting pancreatic cancer stem cell differentiation by lncRNA AB209630/miR-373/EphB2-NANOG signaling pathway.

## Introduction

Pancreatic cancer is one of the most common malignant tumors in the digestive system [1]. It is difficult to detect in the early stage, and it usually advanced when diagnosed. At present, 85% of pancreatic cancer patients die within 1 year after diagnosis, and the 5-year survival rate is less than 50% [2]. Most of them cannot be resected because of invasion or metastasis to extra pancreatic organs, while the resection rate is only about 20% [3]. Pancreatic cancer mainly originates from pancreatic ductal cell malignancies, which are more common in people aged 50–60, and the incidence of male is higher than that of female [4]. In recent years, the incidence of pancreatic cancer has shown a steady upward trend at home and abroad – an increase of about 15% every 10 years. In clinic, about 80% of pancreatic cancer patients have metastasis when symptoms appear [3,4]. Some data show that the incidence of pancreatic cancer in the world is about 185,000 per year, while the incidence of pancreatic cancer in China has increased fourfold in 20 years, reaching 51/100,000 [5].

At present, the adjuvant treatment of pancreatic cancer is disappointing, chemotherapy is still the main treatment, but most of the drug efficacy is poor, chemotherapy is insensitive, side effects are large, the prognosis is very poor [6]. The response rate of chemotherapeutics with relatively good efficacy was only 25%, and the clinical efficacy of gemcitabine was only 28%, and the combined chemotherapy could not improve the efficacy [7–9]. Pancreatic cancer has become a major diagnostic and therapeutic problem faced by the medical community at home and abroad. Any single treatment method is difficult to achieve satisfactory results [4,10]. At the same time, adjuvant chemotherapy is used in order to improve the quality of life and prolong the survival time. Studies have confirmed that adjuvant chemotherapy prolongs the survival time of advanced pancreatic cancer than the best supportive treatment. Therefore, it is necessary to strengthen the comprehensive treatment to improve the current treatment status.

Qingyihuaji formula (QYHJ), a novel Chinese patent medicine, is produced by professor Luming Liu (Department of Integrative Oncology, Fudan University Shanghai Cancer Center, China). QYHJ is produced based on the mechanism theory of ‘damp-heat accumulation’ in pancreatic cancer and innovatively put forward the ‘clearing away heat and removing dampness’ as the main principle for the treatment of advanced pancreatic cancer [11,12]. QYHJ consisting of five traditional Chinese herbs: Banzhilian (Herba *Scutellariae barbatae*), Baihuasheshecao (Herba *Hedyotdis*), Tiannanxing (Rhizoma *Arisaematis erubescentis*), Jiaogulan (Herba seu Radix *Gynostemmatis pentaphylli*), and Doukou (Fructus Amomi Rotundus), which has been used for integrative treatment of human pancreatic cancer for many years [13,14]. While the previous clinical research indicates that QYHJ treatment combined with conventional western medicine has resulted in prolonged survival time for patients with pancreatic cancer [15,16]. In addition, both clinical and animal studies show a response advantage of QYHJ treatment in advanced pancreatic cancer with liver metastases [17]. However, the antitumor effect and the underlying mechanism of QYHJ on gemcitabine resistant human pancreatic cancer remains no any knowledge until now.

In the present study, we aimed to explore and elucidate the antitumor effect and the possible mechanism of QYHJ on gemcitabine resistant human pancreatic cancer in experimental animal *in vivo* and provide more experimental/theoretical evidence for further clinical application of QYHJ.

## Methods and materials

### Materials and reagents

Human pancreatic cancer cell line CFPAC-1 was obtained from American type culture collection (ATCC, Manassas, VA, U.S.A.). Human normal pancreatic cell line hTERT-HPNE was supported by professor Jian Huang (Department of Gastroenterology, Affiliated Hospital of Weifang Medical University). MTT [3-(4,5- dimethyl-2-thiazolyl)-2,5-diphenyl-2-H-tetrazolium bromide, No. G1121] was obtained from Promega. (San Luis Obispo, CA, U.S.A.). Anti-CD133 (ab19898), anti-Ki67 (ab15580) were purchased from Abcam. Caspase-3 antibody (CST 9662) was purchased from Cell Signaling Technology. Gemcitabine (Eli Lilly, Indianapolis, IN, U.S.A.). QYHJ was prepared by the technician, worked in the department of Traditional Chinese Medicine, Affiliated Hospital of Weifang Medical University.

### Preparation of QYHJ extract

QYHJ contains of five traditional Chinese herbs: Banzhilian 30 g, Baihuashe- shecao 15 g, Tiannanxing 15 g, Jiaogulan 30 g, and Doukou 3 g. The five herbs were obtained from Jiang Yin Tianjiang Pharmaceutical Co., Ltd. (Jiangsu, China), the specimens were stored at the department of Traditional Chinese Medicine, Affiliated Hospital of Weifang Medical University (Specimens no: QYHJ2017). The herbs were identified by Professor Peng Chen (Department of Traditional Chinese Medicine, Affiliated Hospital of Weifang Medical University, China).

The five herb mixtures were first soaked in ten volume of distilled water (v/w) for 30 min at the temperature, then boiled for 1 h, and collect the supernatant. The residue was added eight volume of distilled water (v/w) and extracted again. Combined the supernatant and concentrated to 4 g/ml (equal to the weight of dry raw herbs) and then stored at 4°C for use. Gemcitabine dissolved in sterile PBS before use.

### Cell line culture

Human pancreatic cancer cell line CFPAC-1 was cultured in Iscove’s Modified Dulbecco’s Medium (IMDM) with 10% FBS and 1% penicillin–streptomycin. Normal human hTERT-HPNE cells was cultured in DMEM with 5% FBS, 10 ng/ml human recombinant EGF, 5.5 mM D-glucose (1g/l) and 750 ng/ml puromycin. Cells were passaged in every 3 days and cultured in 37°C, 5% CO_2_ humidification incubator. The phase of logarithmic cells was digested by 0.25% trypsin, cell numbers were counted and adjusted to 1 × 10^7^/ml by vaccination for experiment use.

### Cell migration detection

The phase of logarithmic cells of human CFPAC-1 was digested with 0.25% trypsin, diluted to the concentration of 1 × 10^5^/ml, and then took 1 ml cell suspension to each well of 24-well plate. After cell density reach about 60%, used 20 μl of pipet tips to make a line, then changed fresh medium, added the related drugs, and imaged with microscope as the initial width. After the drug treated for 48 h, imaged again and calculated the migration distance.

### Cell invasion and colony formation detection

The phase of logarithmic cells of CFPAC-1 digested and diluted to 1 × 10^5^/ml with the invasion medium of IMDM + 0.1% BSA without serum. About 100 µl of CFPAC-1 cells was added on top of the transwell membrane in the upper chamber and 600 µl of IMDM + 10% FBS + the related drugs was added to the lower chamber in 24-well plate. After drug treatment for 48 h, stained with 0.4% trypan blue and imaged.

The colony formation experiment was detected in 12-well plate. The phase of logarithmic cells of CFPAC-1 digested and diluted to 1 × 10^4^/ml with the complete medium, then seeded the cells to each well with the concentration of 4000 cells/well. After the cell attached, added the related drugs for 48 h incubation, then stained with 0.4% trypan blue, and imaged.

### Animals

About 33 male nude/nude mice (body weight 20 ± 2 g) were supported by the experimental animal center of the Weifang Medical University (Weifang, China). All the animals were reared in SPF grade animal rooms with the temperature of 24 ± 1°C and the humidity of 50 ± 5% followed by 12-h day/night cycles. The animals had free access to food and water, and quarantined for 1 week before experiment. Six mice were raised in one polyacrylic cage in the terms of National Institutes of Health Guidelines of the U.S.A. (National Research Council of U.S.A., 1996) and the University ethical regulations of Weifang Medical University.

### Experimental design

The phase of logarithmic cells of human pancreatic cancer cell line CFPAC-1 was digested and adjusted to 1 × 10^7^/ml, the diluted cells in cell medium were mixed with matrigel with the ratio of 1:1 on ice, take 150 μl of the cell mixture seeded into the right axilla of nude mice with the cell numbers of 1.5 × 10^6^/mice. The xenograft mice were randomly divided into four groups: vehicle group, GEM (35 mg/kg) group, QYHJ (40 g/kg) group [15], GEM combined QYHJ (35 mg/kg + 40 g/kg) group. The daily dosage of QYHJ for the nude mice was calculated according to the following human-mouse transfer formula: D_m_ = D_h_ × (R_m_/R_h_) × (BW_m_/BW_h_)^2/3^, in the formula D, R and BW represent dosage, shape coefficient and body weight, respectively, m and h represent mouse and human, respectively.

After 8 days of the tumor inoculation, the tumor volume was reached to about 100 mm^3^, the mouse was administrated with vehicle (sterile PBS solution), GEM (35 mg/kg), QYHJ (40 g/kg), and GEM + QYHJ (35 mg/kg + 40 g/kg) through oral administration based on the oral volume of 10 ml/kg BW, once a day, respectively. All the animals were killed by cervical dislocation in 28th days at the end of the experiment. The tumor tissues of all the xenografts were harvested and weighted, and all the tumor tissue were quick freezing in liquid nitrogen, and stored at liquid nitrogen tank for use.

### Quantitative real-time PCR

Use TRIzol reagent (15596026, invitrogen, Shanghai, China) to extract the total RNA samples of each tumor tissue, according to the manufacture’s introduction. Take 1 μg of total RNA to reverse transcribed cDNA using a MiRcute miRNA First-strand cDNA synthesis kit (Tiangen Biotech, Beijing, China) for miRNA, while the Primer-Script TM one step real-time PCR reagent kit (Takara, Shiga, Japan) was used for the reverse transcription of lncRNAs and mRNAs, according to the manufacturer’s protocol. The relative quantitation of mRNA expression levels was measured using the SYBR Green I real-time PCR kit (CoWin Bioscience Co., Beijing, China). The sequences of all the primer were lncRNA AB209630 (90 bp): forward 5′-GGGCTATTGTCCCTAAGTTGAT-3′, reverse 5′-TGTCTTGTAGAGCATAAGG AAACC-3′; miR-373: forward 5′-ACUCAAAAUGGGGGCGCUUUCC-3′, reverse 5′-GAAGUGCUUCGAUUUUGGGGUGU-3′; EphB2 (149 bp) forward 5′-CAGGTACATATCACGCGCACAG-3′; reverse 5′-TGTAAACAAACCCAGATG CAGGA-3′; NANOG (403 bp) forward 5′-ATGCCTGTGATTTGTGGGCC-3′; reverse 5′-GCCAGTTGTTTTTCTGCCAC-3′; GAPDH (353 bp) forward 5′-GGGAGCCAAAAGGGTCATCATCTC-3′; reverse 5′-CCATGCCAGTGAGCTT CCCGTTC-3′. In order to normalize the gene expression levels in each tumor tissue sample, the gene of GAPDH was used as an endogenous control for lncRNA and mRNAs, whereas U6 was used as internal reference for miRNAs. The changes of the mRNA expression in all the groups were calculated by the method of 2^−ΔΔ*C*^_T_ [18].

### Tumor sphere culture

Take proper amount of fresh tumor tissue and wash with PBS solution. Mince the tumor tissues and digest it with 1 mg/ml collagenase IV for 1 h at 37°C. Pipet the suspension every 20 min, observe it under the microscope. Then add DMEM/F12 complete medium (containing 10% FBS) to terminate the digestion. Strain cells through a 40-μm sieve. Harvest cells by centrifugation in 15 ml tube (1000 rpm for 10 min) and discard the supernatant. Wash cells with PBS solution. Dilute single cell suspension to 1 × 10^6^–10^7^ cells/ml and plant the cell suspension to the ultra-low adherent six-well plate with 2 ml per well. Culture the cell under incubator with the condition of 37°C, 5% CO_2_.

### Statistical analysis

The values presented in the study were represented as mean ± S.D. One-way ANOVA test followed by Dunett’s t-test was used as a calculated statistical method with SPSS19.0 statistical software. *P*<0.05, *P*<0.01, and *P*<0.001 were regarded as statistically significant.

## Results

### Cell proliferation inhibitory effect of QYHJ on GEM resistant CFPAC-1 cell line

In order to detect the antitumor effect of QYHJ, we first measured the cell proliferation inhibitory effects of QYHJ on CFPAC-1 pancreatic cancer cell line *in vitro*. From Table 1, we could see that QYHJ could significantly increase the cell proliferation inhibitory effect in the dosage of 40 μg/l with the extension of time (*P*<0.05, *P*<0.01, *P*<0.001). GEM only in the time of 72 h could inhibit the cell proliferation (*P*<0.05). More interestingly, in the combination of GEM and QYHJ treatment group, cell proliferation inhibitory effects were dramatically increased in the longer of the treatment times (24, 48, and 72h) than GEM or QYHJ alone treatment, which implied that QYHJ could enhance the activity of GEM in the pancreatic cancer cell. In addition, we also detected the cell proliferation inhibition of QYHJ in normal hTERT-HPNE cells, the result indicated that there has a slightly cell proliferation inhibition, but no significant difference compared with the control group (Supplementary Figure S1).

**Table 1:** Cell proliferation inhibitory effect of QYHJ on CFPAC-1 cell line

Group	Dosage(ng/l)	Inhibitory ratio %		
		24 h	48 h	72 h
**Control**	-	0.0	0.0	0.0
**GEM**	30 ng/l	13.1 ± 1.3	34.2 ± 2.1*	51.7 ± 4.0**
**QYHJ**	40 μg/l	35.6 ± 1.9*	52.3 ± 3.2**	73.5 ± 4.8***
**GEM+QYHJ**	30 ng/l + 40 μg/l	54.4 ± 3.3**	70.9 ± 4.3**	93.3 ± 5.9***

Data are expressed as mean ± S.D. for each group. *P<0.05, **P<0.01, ***P<0.001 vs control group.

### Cell apoptosis effect of QYHJ on GEM resistant CFPAC-1 cell line

Based on the cell proliferation inhibitory effect, we would like to know if QYHJ could cause the pancreatic cancer cells to apoptosis. Thus, we continue testing the apoptosis changes in GEM alone, QYHJ alone, or the combination of GEM and QYHJ treated CFPAC-1 cell line at the time of 12, 18, and 24 h, respectively (Table 2). The result showed that QYHJ could significantly cause the pancreatic cancer cells to apoptosis in 12, 18, and 24 h, respectively (*P*<0.05, *P*<0.01), compared with the control group while the combination of GEM and QYHJ group markedly promoted the apoptosis phenomena (*P*<0.01, *P*<0.001, Table 2).

**Table 2:** Cell apoptosis assay on CFPAC-1 cell line at the indicated times

Group	Dosage (ng/l)	Apoptosis ratio %		
		12 h	18 h	24 h
**Control**	-	2.8 ± 0.3	4.2 ± 0.8	4.6 ± 0.5
**GEM**	30 ng/l	11.0 ± 1.2	30.0 ± 2.7*	44.3 ± 2.3*
**QYHJ**	40 μg/l	37.1 ± 2.1*	44.1 ± 2.5*	56.1 ± 2.2**
**GEM+QYHJ**	30 ng/l + 40 μg/l	50.6 ± 3.2**	63.9 ± 3.1**	80.9 ± 6.9***

Data are expressed as mean ± S.D. for each group. *P<0.05, **P<0.01, ***P<0.001 vs control group.

### Cell migration detection after QYHJ treatment

**Figure 1:**
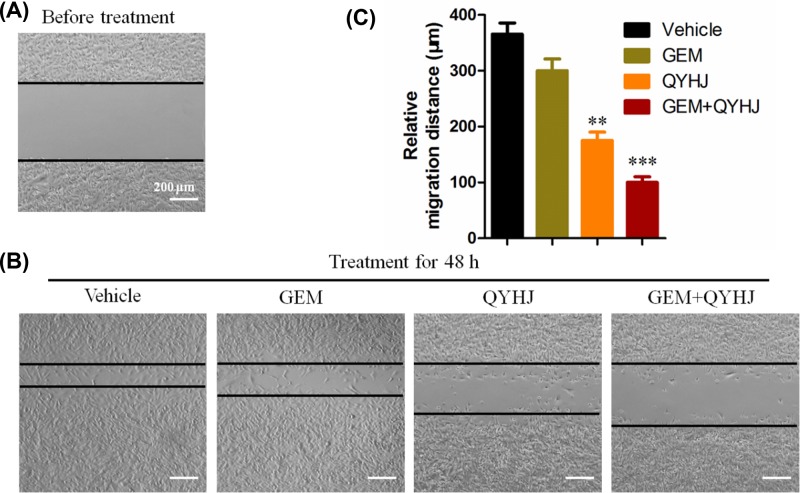
The migration distance detected by the wood-healing experiment in CFPAC-1 cell line under the treatment of vehicle, GEM (30 ng/l), QYHJ (40 μg/l), and GEM + QYHJ (30 ng/l + 40 μg/l) (scale bar 200 μm) for 48 h **P<0.01, ***P<0.001 vs vehicle group.

As we know, pancreatic cancer cells are easily migration to the whole body; here, we detected if QYHJ could inhibit the cell migrations *in vitro*. In the experiment, since the cells are in logarithmic growth phase after attached 48 h, so we chose 48 h as the observation time. As Figure 1 shown, after 48-h treatment, the cells in vehicle group have strong migration ability, which are almost migrated to the center. GEM alone treated cells has a slightly inhibit the cell migration, but there is no significant difference, compare with the vehicle group. QYHJ alone treated cells were significantly decrease the cell migration, compared with the vehicle group (*P*<0.01, Figure 1). More surprisingly, the combination of GEM and QYHJ treated cells were dramatically inhibited the cell migration, compared with the vehicle group (*P*<0.01, Figure 1).

### Cell invasion and colony formation assay after QYHJ treatment

**Figure 2:**
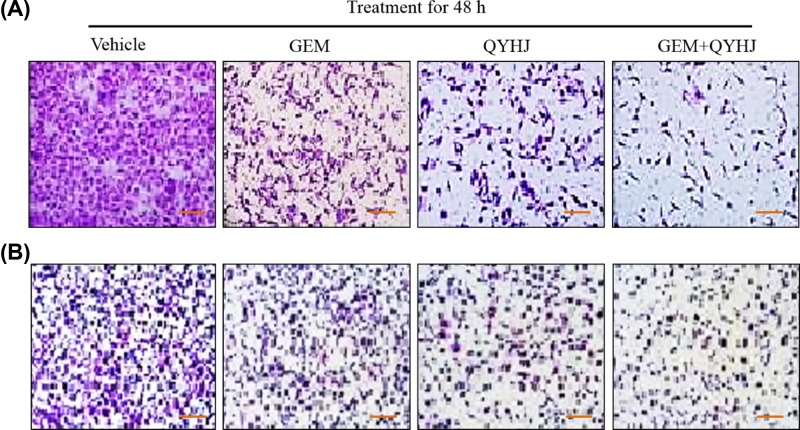
Cell invasion and colony formation experiment in CFPAC-1 cell line under the treatment of vehicle, GEM (30 ng/l), QYHJ (40 μg/l), and GEM + QYHJ (30 ng/l + 40 μg/l) (scale bar 100 μm) for 48 h

In addition, we also assayed cell invasion through transwell experiment and measured the ability of cell colony formation after QYHJ treatment at the same time (Figure 2). Figure 2A showed the cell invasion result, it exhibited that vehicle cells had a strong invasion ability. GEM and QYHJ treated cells dramatically inhibit the cell invasion, compared with vehicle group. While consistent with the result of cell migration, the combination of GEM and QYHJ treated cells were remarkably inhibited the cell invasion, compared with the vehicle, GEM and QYHJ alone group. At the same time, we also detected the colony formation effect in GEM, QYHJ, and the combination of GEM and QYHJ treated CFPAC-1 cells (Figure 2B). It showed a consistent result with the cell migration and cell invasion.

### Effect of QYHJ on GEM resistant CFPAC-1 tumor xenografts

**Figure 3:**
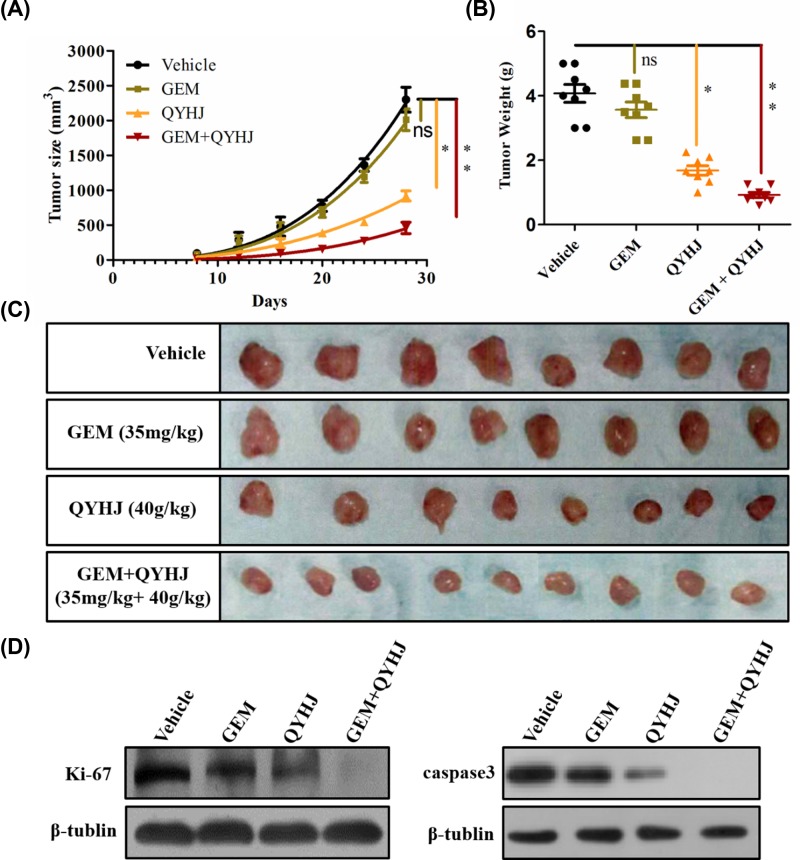
Effects of QYHJ on GEM resistant human pancreatic cancer cell line CFPAC-1 tumor xenografts (**A**) Tumor size, (**B**) tumor weight, and (**C**) tumor morphology photos of vehicle, GEM (35 mg/kg), QYHJ (40 g/kg) and GEM + QYHJ (35 mg/kg + 40 g/kg) group, (**D**) the protein expression of Ki-67 and caspase 3 in tumor tissue of each group. *P<0.05, **P<0.01 vs vehicle control group.

Based on the above result, we continue studying the *in vivo* antitumor effect of QYHJ on GEM resistant CFPAC-1 tumor xenografts. As Figure 3 shown, GEM alone treatment group has almost no antitumor effect, QYHJ alone treated group has a remarkable tumor inhibitory effect *(P*<0.05), while QYHJ significantly promoted the antitumor effect of GEM in the combination of GEM and QYHJ treated group (*P*<0.01, Figure 3A–C), compared with the vehicle group. Figure 3A showed the tumor size changes in different groups along with the experiment period. The result showed that the tumor in the combination of GEM and QYHJ treated group significantly decreased. Figure 3B showed the final tumor weight in all the experiment groups, which also displayed a significantly decrease in the group of GEM combined QYHJ (*P*<0.01), compared with the vehicle group. Figure 3C showed the final tumor morphology from all the experimental mice. In addition, we also detected the tumor cell proliferation and apoptosis effect in tumor tissue by western blot method (Figure 3D). The result showed that GEM combined QYHJ treated group significantly, decreased the tumor cell proliferation, and also significantly promoted the tumor cell apoptosis in tumor tissue (Figure 3D). Furthermore, we did not observe any changes in the body weight of the mice in the whole progress of the experiment (more details see Supplementary Figure S2), and all the animals were survived in the whole experiment, which reflected that QYHJ has a very lower side effects *in vivo* experiment.

### Effect of QYHJ on the mRNA expression levels of GEM resistant tumor tissue

**Figure 4:**
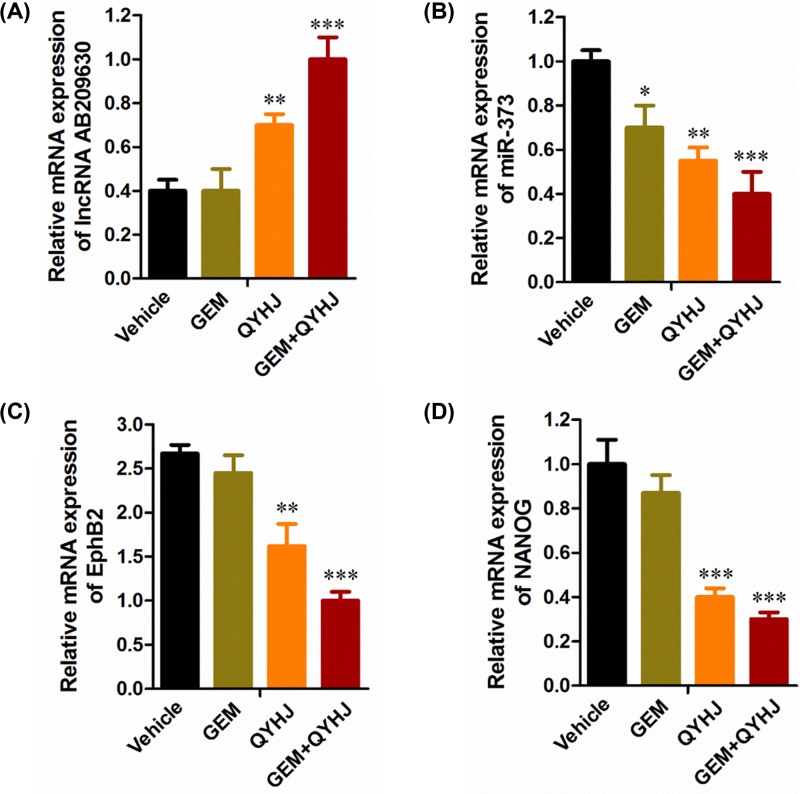
The mRNA expression of lnc RNA AB209630, miR-373, EphB2 and NANOG in human pancreatic cancer CFPAC-1 cell line after QYHJ administration Effects of QYHJ on mRNA expression of lnc RNA AB209630, miR-373 and EphB2, and NANOG genes in human pancreatic cancer CFPAC-1 cell line **(A–D)**. Data are expressed as mean ± S.D. for each group. *P<0.05, **P<0.01, ***P<0.001 vs vehicle group.

In order to determine the mechanism of QYHJ on GEM resistant tumor tissue, we detected the mRNA expression of lncRNA AB209630, miR-373, EphB2, and NANOG in tumor tissue. From Figure 4, we could see that GEM treatment alone almost had no effect on the expression of lncRNA AB209630 and NANOG. QYHJ treatment alone could significantly increase the levels of lncRNA AB209630 and decrease the levels of miR-373, EphB2, and NANOG (*P*<0.01, *P*<0.001, Figure 4A–D). While in the group of the combination of GEM and QYHJ, QYHJ significantly enhanced the sensitivity of GEM on the expression of lncRNA AB209630, and significantly enhanced the inhibitory effect of GEM on the expression of miR-373, EphB2, and NANOG (*P*<0.001, Figure 4A–D). This result gives us a little hint that QYHJ reverses the antipancreatic cancer effect may be through the mechanism of regulation the lncRNA AB209630/ miR-373/EphB2-NANOG signaling pathway in tumor.

### Effect of QYHJ on tumor sphere forming and CD133+ stem cells

**Figure 5:**
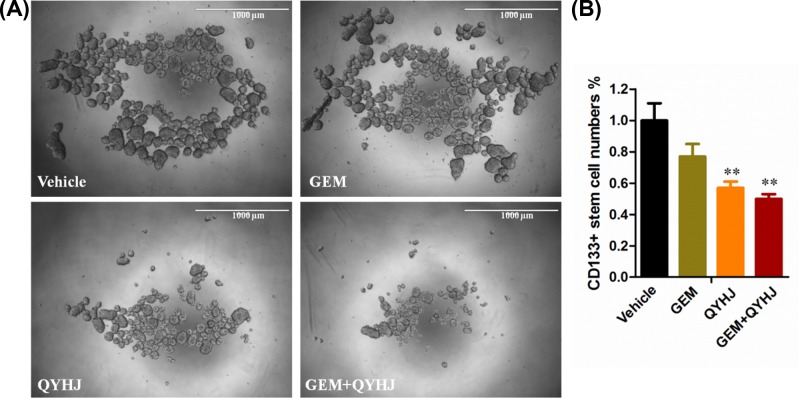
Effect of QYHJ on tumor sphere forming and CD133+ stem cells The tumor sphere forming (**A**) and CD133+ cell numbers from the xenograft tumor (**B**). **P<0.01 vs vehicle group.

As we know, cancer stem cell is the biggest obstacle for tumor resistance; thus, in the study, we detected the effect of tumor sphere forming and CD133+ stem cells in each group. In Figure 5A, it is shown that GEM almost had no effect in inhibiting the tumor sphere formation, while QYHJ treatment alone dramatically decreased the sphere formation, and the combination of the GEM and QYHJ treatment group was also significantly inhibited the tumor sphere formation. Furthermore, we also detected the numbers of the CD133+ stem cells in each group, which showed a same result with the tumor sphere formation. The combination of GEM and QYHJ significantly decreased the numbers of the CD133+ stem cells (*P*<0.01, Figure 5B).

## Discussion

Pancreatic cancer is a common lethal cancer of digestive system. Surgery is the only treatment for long-term survival of patients with pancreatic cancer. Only 10–15% of patients have the chance of surgical resection [5]. The 5-year survival rate after surgery is about 20%. Patients often give up radical operation because of intraoperative metastasis or macrovascular invasion. Therefore, nonsurgical treatment occupies the main position in the comprehensive treatment of pancreatic cancer. Nonsurgical treatment of pancreatic cancer includes chemical drug therapy, radiotherapy, physiotherapy, traditional Chinese medicine treatment, and molecular targetted therapy. Chemotherapy is the main treatment for advanced pancreatic cancer or metastatic pancreatic cancer. GEM-based chemotherapy regimens have been widely used in clinic. GEM combined with capecitabine can improve the half-year survival rate and objective remission rate of patients [8]. It supports being the first line of advanced pancreatic cancer. However, some patients have been resistant to GEM, and there is no unified second-line treatment plan [7].

Qingyihuaji formula (QYHJ), a novel Chinese patent medicine, has been reported that it has a strong antitumor effect in pancreatic cancer cells. Here, we wonder that if QYHJ has the effect of reverse GEM induced cancer resistance, if yes, what the action mechanism is. In the present study, from *in vitro* cell proliferation, cell apoptosis, and cell migration experiment, we could see that QYHJ displayed a significant inhibition in GEM resistant cell proliferation, cell, and also displayed a promoted cell apoptosis effect on GEM resistant pancreatic cancer cells.

IncRNA is a hot topic in the field of noncoding RNA. It is also a hot topic in the field of oncology. A genomic transcription analysis including microarray analysis and sequencing analysis showed that many tumor-related lncRNAs were clearly identified, indicating that lncRNAs have a close relationship with tumors, which provides a lesson for our research [19]. A large number of studies have found that lncRNAs play a new role in the mechanism of tumorigenesis and play a regulatory role in both carcinogenic and anticarcinogenic expression pathways of tumors [20]. Currently, many important tumors lncRNA are known to exist in malignant tumors of many human organs, such as PCGEM1, ANRIL, DD3 in prostate cancer, HOTAIR in breast cancer, colorectal cancer, and laryngeal cancer, XIST in breast cancer, testicular cancer, MLAT1, Neat2 in early nonsmall cell lung cancer. HULC is present in laryngeal and colorectal cancers. While lncRNA AB209630 reported as an oncogene, down expression in many tumors, which can used as a new biomarker for tumorigenesis. Furthermore, decreased expression of AB209630 in patients would have a significantly poorer prognosis than those with high AB209630 expression [21–23]. Thus, increase the expression of lncRNA AB209630 may be a new therapeutic strategy for tumor. In the present study, we found that QYHJ treated mouse significantly increased the mRNA expression of lncRNA AB209630, which significantly promote the inhibitory effect of GEM in the xenografts.

With the discovery of new miRNAs and the elucidation of some functions of miRNAs, the research of miRNAs has attracted much attention and become one of the hotspots of current research [24,25]. The role of miRNAs in tumors is mainly in angiogenesis, apoptosis, invasion, metastasis, cell proliferation, and tumorigenesis. MiRNA-373 is a potential new oncogene. It can reduce the sensitivity of cells to the effect of *p*53 allele expressed normally by blocking the ageing, induced by RAS and interfering with RAS-mediated cell transformation. It can block the CDK inhibition pathway mediated by *p*53 or directly inhibit the expression of LATS2, thus promoting the tumor cell formation of testicular embryos [26]. These studies show that miRNA-373 is closely related to tumor occurrence and tumor cell proliferation. In the present study, QYHJ dramatically improved the inhibitory effect of GEM on GEM resistant pancreatic cancer xenografts.

Up to now, members of Eph family have been identified to include at least 14 receptors and eight ligands. EphB2 is one of the members of this subgroup [27,28]. EphB2 is mainly participating in the phosphorylation of amino acid, protein kinase signal transduction of transmembrane receptor, nervous system development, and cancer progression [29,30]. Abnormal expression of EphB2 receptor has been found in many kinds of tumors. Batlle et al. [31] detected the expression of EphB2 receptor in normal crypt stem cells, intestinal adenomas, *in situ* intestinal carcinoma, lymph node metastasis of intestinal cancer and hepatic metastasis, and found that the expression of EphB2 was decreased. Oshima et al. [28] pointed out that the low expression of EphB2 is closely related to liver metastasis of colon cancer, which may be an important prognostic factor in patients with liver metastasis of colon cancer. However, the study of EphB2 in other tumors has drawn completely different or even opposite conclusions. Nakada et al. [32,33] showed that EphB2 was highly expressed in prostate cancer, gastric cancer, breast cancer, small cell lung cancer, and pancreatic cancer, but not in normal tissues. Our study showed that EphB2 highly expressed in the tissue of pancreatic cancer tumors, and QYHJ displayed a strong effect on the decrease of the expression of EphB2. Furthermore, QYHJ also enhanced the inhibitory effect of GEM on EphB2 expression.

NANOG is a new transcription factor found in embryonic stem cells by Chambers and Mitsui in 2003. It belongs to the ANTP-like NK family gene. It is located on the chromosome 12, 12p13.31, with a total length of 6661 bp. It consists of four exons and three introns, and encodes 305 amino acids. It is the key genes for stem cells on maintain self-proliferation and multidirectional differentiation [34–36]. With the development of research, NANOG has been found highly expression in breast cancer, spermatogonioma, gastric cancer, intestinal cancer, cervical cancer, oral squamous cell cancer and pancreatic cancer, and so on. And the expression of NANOG is closely related to the occurrence, development and prognosis of these tumors [37]. The results in the present study exhibited that QYHJ has a significant inhibitory effect in the mRNA expression of NANOG, which implied that the inhibition effect of QYHJ may be related with the cancer stem cell.

Thus, in our study, we detected the influence of QYHJ in cancer stem cells (CSCs) in GEM resistant pancreatic cancer xenografts. As we know, CSCs are responsible for tumor initiation and propagation, which also plays a vital role in predicting prognosis in cancer patients, high levels of CSCs lead to poor prognosis and targetting CSCs results in prolonged survival [6,38,39]. Tumor sphere culture in low-adherent plate is one of the enrich methods for CSCs, while CD133+ cells are defined as pancreatic CSCs [40]. Thus, in our study, the data showed that QYHJ significantly reduced the tumor sphere forming and decreased the number of CD133+ cells, which also promoted the inhibition effect of GEM.

In conclusion, QYHJ exhibited a significant effect against the GEM resistance pancreatic cancer, which may be probably through the inhibition of cell migration, up-regulate the levels of lncRNA AB209630 and down-regulate the levels of miR-373, EphB2, and NANOG. More importantly, QYHJ decreases the forming ability of CSCs, which may be the main mechanism for QYHJ to prolong the patient life in clinical. The present study implied that QYHJ could be an alternative for GEM resistant pancreatic cancer patients.

## Availability of data and materials

All data generated or analyzed during the present study are included in this published article and its supplementary information files.

## Abbreviations

BW: body weight
BSA: bovine serum albumin
CSC: cancer stem cell
GAPDH: glyceraldehyde-3-phosphate dehydrogenase
GEM: gemcitabine
IMDM: Iscove’s Modified Dulbecco’s Medium
PI: propidium iodide
QYHJ: Qingyihuaji formula
TS: tumor size
TW: tumor weight
